# The crystal structures of two new coumarin derivatives: 2-(4-{2-[(2-oxo-2*H*-chromen-4-yl)­oxy]acet­yl}piperazin-1-yl)acetamide and *N*-(2,4-di­meth­oxy­benz­yl)-2-[(2-oxo-2*H*-chromen-4-yl)­oxy]acetamide

**DOI:** 10.1107/S2056989019003736

**Published:** 2019-03-26

**Authors:** S. Syed Abuthahir, M. NizamMohideen, V. Viswanathan, M. Govindhan, K. Subramanian

**Affiliations:** a PG & Research Department of Physics, The New College (Autonomous), Chennai 600 014, Tamil Nadu, India; b Department of Biophysics, All India Institute of Medical Science, New Delhi 110 029, India; c Department of Chemistry, Anna University, Chennai 600 025, India; d Orchid Chemicals & Pharmaceuticals Ltd, R&D Centre, Sholinganallur, Chennai 600 119, India

**Keywords:** crystal structure, chromen, piperazine, acetamide, pyran, hydrogen bonding, C—H⋯π inter­actions, offset π–π inter­actions, Hirshfeld surface analysis

## Abstract

The title compounds, 2-(4-{2-[(2-oxo-2*H*-chromen-4-yl)­oxy]acet­yl}piperazin-1-yl)acetamide, C_17_H_19_N_3_O_5_, (I), and *N*-(2,4-di­meth­oxy­benz­yl)-2-[(2-oxo-2*H*-chromen-4-yl)­oxy]acetamide, C_20_H_19_NO_6_, (II), are new coumarin derivatives. In compound (I), the six-membered piperazine adopts a chair conformation. The dihedral angles between the mean planes of the chromene ring and amide plane is 82.65 (7)° in (I) and 26.2 (4)° in (II). The dihedral angles between the mean planes of the chromene ring and the four planar C atoms of the piperazine ring in (I) and the benzene ring in (II) are 87.66 (6) and 65.0 (4)°, respectively. There are short intra­molecular contacts in both mol­ecules forming *S*(5) ring motifs, *viz*. N—H⋯N and C—H⋯O in (I), and N—H⋯O and C—H⋯N in (II). In the crystals of both compounds, mol­ecules are linked by N—H⋯O hydrogen bonds, forming chains along [10] in (I) and [010] in (II). The chains are linked by C—H⋯O hydrogen bonds, forming layers parallel to the *ab* plane in the crystals of both compounds. In the crystal of (I), there are also C—H⋯π and offset π–π inter­actions [inter­centroid distance = 3.691 (1) Å] present within the layers. In the crystal of (II), there are only weak offset π–π inter­actions [inter­centroid distance = 3.981 (6) Å] present within the layers. The inter­molecular contacts in the crystals of both compounds have been analysed using Hirshfeld surface analysis and two-dimensional fingerprint plots.

## Abstract 2

The crystal structures of two 2-[(2-oxo-2*H*-chromen-4-yl)­oxy]acetamide derivatives are described and the inter­molecular contacts in the crystals analysed using Hirshfeld surface analysis and two-dimensional fingerprint plots.

## Chemical context   

Coumarin and its derivatives represent one of the most active classes of compounds possessing a wide spectrum of biological activity. The synthesis, and pharmacological and other properties of coumarin derivatives have been studied and reviewed (Kumar *et al.*, 2015 ▸; Kubrak *et al.*, 2017 ▸; Srikrishna *et al.*, 2018 ▸; Venugopala *et al.*, 2013 ▸). Many of these compounds have proven to be active as anti­bacterial, anti­fungal, anti-inflammatory, anti­coagulant, anti-HIV and anti­tumor agents. One of the title compounds, 2-(4-{2-[(2-oxo-2*H*-chromen-4-yl)­oxy]acet­yl}piperazin-1-yl)acetamide (I), has been shown to exhibit anti­microbial as well as anti­oxidant activity (Govindhan, Subramanian, Chennakesava Rao *et al.*, 2015 ▸; Govindhan, Subramanian, Sridharan *et al.*, 2015 ▸). In view of the importance of their natural occurrence, biological activities, pharmacological and medicinal activities, and utility as synthetic inter­mediates, we have synthesized the title 2-[(2-oxo-2*H*-chromen-4-yl)­oxy]acetamide derivatives, and report herein their crystal structures and Hirshfeld surface analysis.
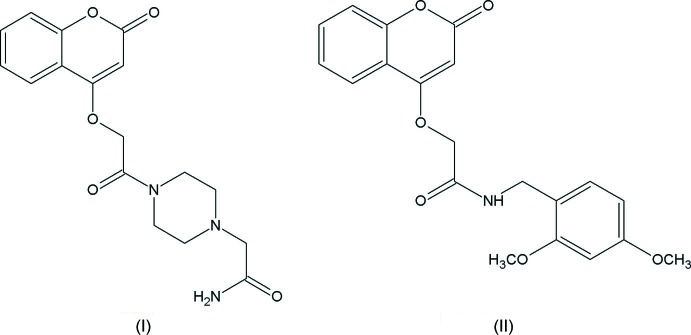


## Structural commentary   

**Figure 1:**
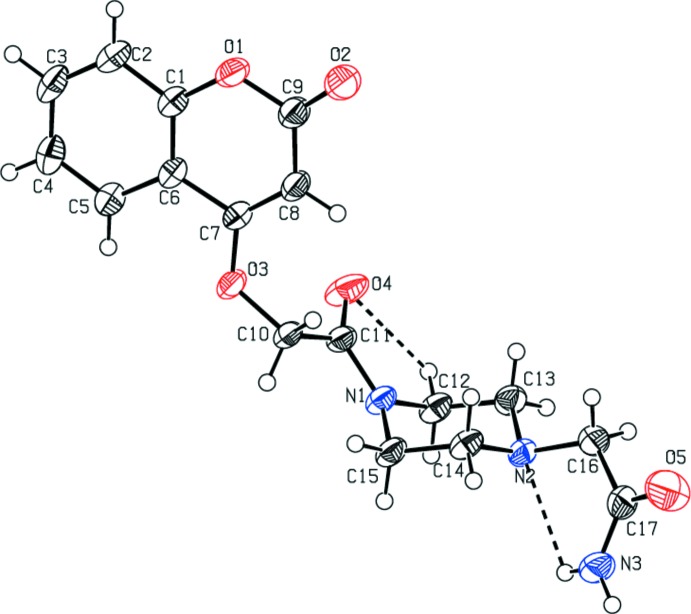
The mol­ecular structure of compound (I), with the atom labelling. Displacement ellipsoids are drawn at the 50% probability level. Intra­molecular contacts (Table 1 ▸) are shown as dashed lines.

The mol­ecular structures of compounds (I) and (II) are illus­trated in Figs. 1 ▸ and 2 ▸, respectively. In (I), the piperazine ring (N1/N2/C12–C15) is attached to the 2-[(2-oxochroman-4-yl)­oxy]acetaldehyde moiety on atom N1 and to an acetamide moiety on atom N2. It has a chair conformation [puckering parameters: total amplitude *Q* = 0.561 (2) Å, θ = 0.67 (2)° and φ = 149 (2)°], and is positioned *anti* with respect to the C—N rotamer of the amide. Nevertheless, because the asymmetry of the chromene residue, the *anti* conformation can assume a *cis* or *trans* geometry with respect to the relative position of the carbonyl O atom of the carboxamide and the C10—C11 and C16—C17 bonds. Both compounds exhibit a *cis* relation between these bonds, as can be seen in Figs. 1 ▸ and 2 ▸. This mol­ecular conformation permits the formation of intra­molecular hydrogen bonds (Tables 1 ▸ and 2 ▸), which enhance the relative planarity of each compound. Specifically for each compound, as a result of the presence of the imidic nitro­gen atom, the mol­ecules display intra­molecular N—H⋯N and N—H⋯O hydrogen bonds, between the amide nitro­gen and the nitro­gen atom N2 of the piperazine ring for compound (I), and oxygen atom O3 for (II), forming *S*(5) ring motifs. In addition, the carbonyl oxygen atom O4 acts as the acceptor for a weak inter­action with a hydrogen bond of the exocyclic piperazine ring, forming a second *S*(5) ring motif in (I), and the amide nitro­gen atom N1 acts as the acceptor for a weak inter­action with a hydrogen bond of the exocyclic benzene ring, forming a second *S*(5) ring motif in (II).

The values of the dihedral angles between the mean planes of the planar chromene ring system (O1/C1–C9; r.m.s. deviations = 0.008 Å for both compounds) and the amide plane (C10/C11/O4/N1) are 82.65 (7) and 26.2 (4)° in compounds (I) and (II), respectively. In (I), the dihedral angle between the mean planes of the chromene ring and the four C atoms (C12–C15) of the piperazine ring is 87.66 (6)°, while in (II) the benzene ring (C13–C18) is inclined to the mean plane of the chromene ring by 65.0 (4)°. Atom O2 deviates from the coumarin ring mean plane by 0.051 (1) Å in (I) and −0.043 (9) Å in (II).

It is inter­esting to compare the intra­molecular hydrogen bonding present in the title compounds with that of the analogous 4-oxo-*N*-(substituted phen­yl)-4*H*-chromene-2-carboxamides (Reis *et al.*, 2013 ▸; Gomes *et al.*, 2013 ▸). It can be seen that the effect of the 2/3 positional isomerism is to ‘reflect’ their relative positions while the effect of the *cis/trans* conformations is a ‘twofold rotation’ of the rings around the C_amide_—C_chromene_ bond. These particular differences in conformation may condition the ability for docking when pharmacological activities are considered.

## Supra­molecular features   

**Figure 3:**
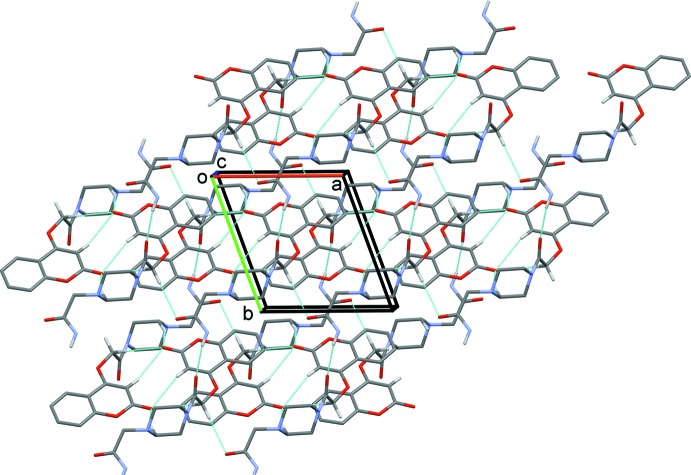
A view along the *c* axis of the crystal packing of compound (I). The hydrogen bonds (Table 1 ▸) are shown as dashed lines, and H atoms not involved in hydrogen bonding have been omitted.

In the crystal of (I), mol­ecules are linked by N3—H3*A*⋯O4^i^ hydrogen bonds, forming chains along the [1
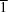
0] direction, see Fig. 3 ▸ and Table 1 ▸. The chains are linked by C—H⋯O hydrogen bonds, forming layers lying parallel to the *ab* plane (Fig. 3 ▸ and Table 1 ▸). The C14—H14*A*⋯O2^ii^ hydrogen bond generates an inversion dimer with an 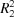
(22) ring motif; within the ring C8—H8⋯O2^ii^ and C10—H10*B*⋯O2^ii^ hydrogen bonds link the mol­ecules into 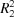
(8) and 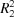
(14) rings, respectively. These rings are linked by *C*(10) and *C*(7) chains formed *via* the C10—H10*A*⋯O5^iii^ and N3—H3*A*⋯O4^i^ hydrogen bonds, respectively. A C—H⋯π inter­action is also present within the layer (Table 1 ▸). An offset π–π contact between inversion-related chromene rings further stabilizes the crystal structure [*Cg*2⋯*Cg*2^iv^ = 3.691 (1) Å, inter­planar distance = 3.490 (1) Å, offset = 1.20 Å; *Cg*2 is the centroid of the O1/C1–C9 ring; symmetry code: (iv) −*x* + 1, −*y* + 1, −*z* + 1].

**Figure 4:**
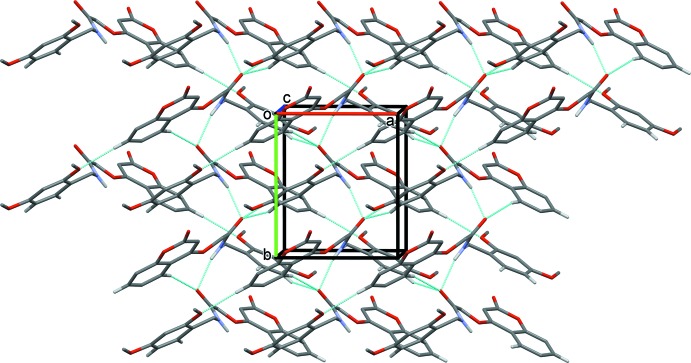
A view along the *a* axis of the crystal packing of compound (II). The hydrogen bonds (Table 2 ▸) are shown as dashed lines, and H atoms not involved in hydrogen bonding have been omitted.

In the crystal of (II), mol­ecules are linked by N1—H1⋯O4^i^ hydrogen bonds, forming chains along the [010] direction, see Fig. 4 ▸ and Table 2 ▸. The chains are linked by C3—H3⋯O5^ii^, C5—H5⋯O4^i^ and C15—H15⋯O4^iii^ hydrogen bonds, forming layers parallel to the *ab* plane (Fig. 4 ▸ and Table 2 ▸). Within the layer there are no C—H⋯π inter­actions present, only weak offset π–π inter­actions involving the benzene ring of the chromene ring system and the di­meth­oxy­benzene ring [*Cg*2⋯*Cg*3^iv^ = 3.981 (6) Å, inter­planar distances = 3.638 (4) and 3.508 (4) Å, offset 0 1.88 Å; *Cg*2 and *Cg*3 are the centroids of rings C1–C6 and C13–C18, respectively; symmetry code: (iv) −*x* + 1, *y* + 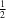, −*z* + 1].

## Hirshfeld surface analysis   

A recent article by Tiekink and collaborators (Tan *et al.*, 2019 ▸) reviews and describes the uses and utility of Hirshfeld surface analysis (Spackman & Jayatilaka, 2009 ▸), and the associated two-dimensional fingerprint plots (McKinnon *et al.*, 2007 ▸), to analyse inter­molecular contacts in crystals. The various calculations were performed with *CrystalExplorer17* (Turner *et al.*, 2017 ▸).

**Figure 5:**
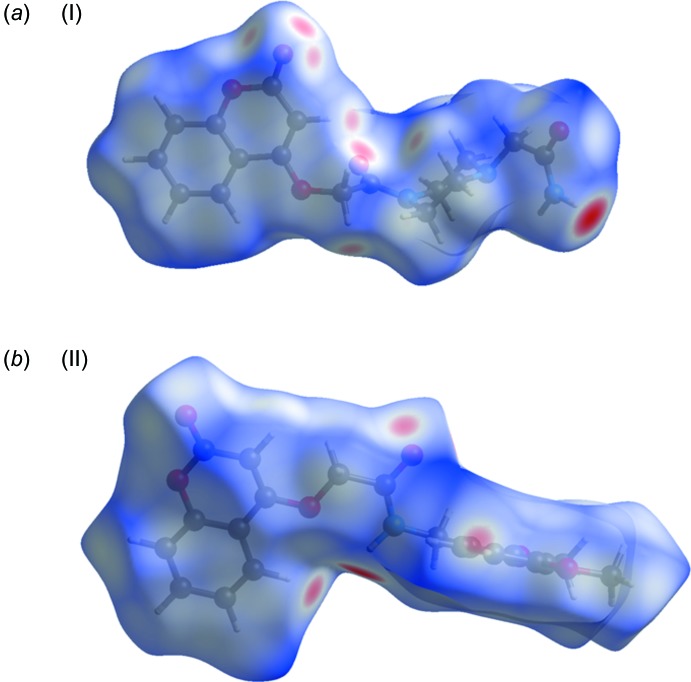
The Hirshfeld surfaces of compounds (*a*) (I) and (*b*) (II), mapped over *d*_norm_

**Figure 6:**
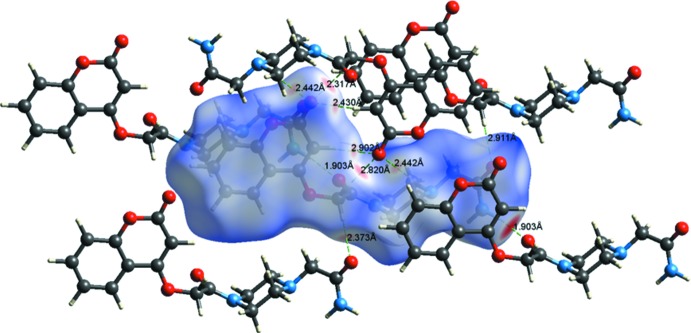
A view of the Hirshfeld surface mapped over *d*_norm_ of compound (I), showing the various inter­molecular contacts in the crystal.

**Figure 7:**
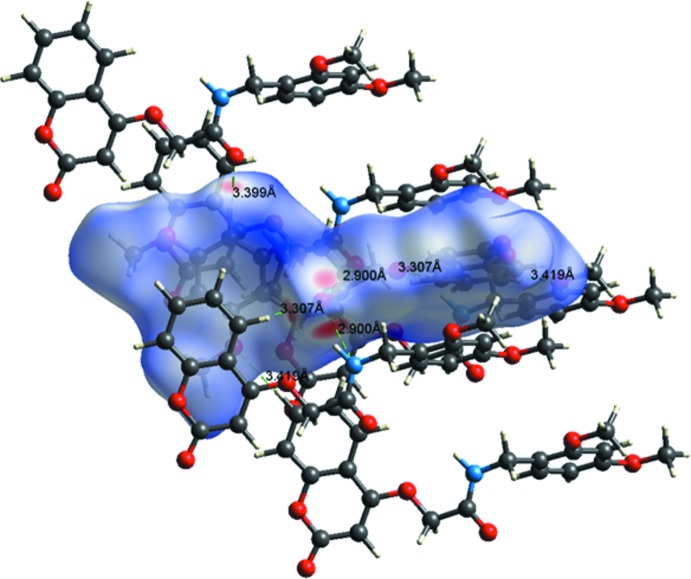
A view of the Hirshfeld surface mapped over *d*_norm_ of compound (II), showing the various inter­molecular contacts in the crystal.

The Hirshfeld surfaces of compounds (I) and (II) mapped over *d*_norm_ are given in Fig. 5 ▸, and the inter­molecular contacts are illustrated in Fig. 6 ▸ for (I) and Fig. 7 ▸ for (II). They are colour-mapped with the normalized contact distance, *d*_norm_, from red (distances shorter than the sum of the van der Waals radii) through white to blue (distances longer than the sum of the van der Waals radii). The *d*_norm_ surface was mapped over a fixed colour scale of −0.544 (red) to 1.418 (blue) for compound (I) and −0.501 (red) to 1.672 (blue) for compound (II), where the red spots indicate the inter­molecular contacts involved in the hydrogen bonding.

**Figure 8:**
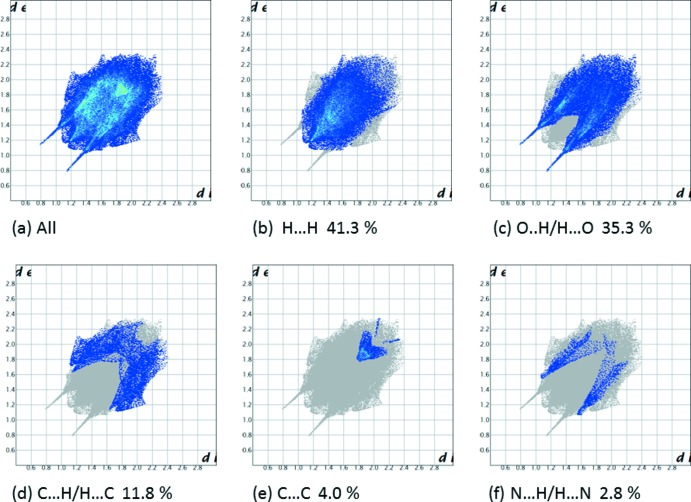
The full two-dimensional fingerprint plot for compound (I), and fingerprint plots delineated into (*b*) H⋯H, (*c*) O⋯H/H⋯O, (*d*) C⋯H/H⋯C, (*e*) C⋯C and (*f*) N⋯H/H⋯N contacts.

**Figure 9:**
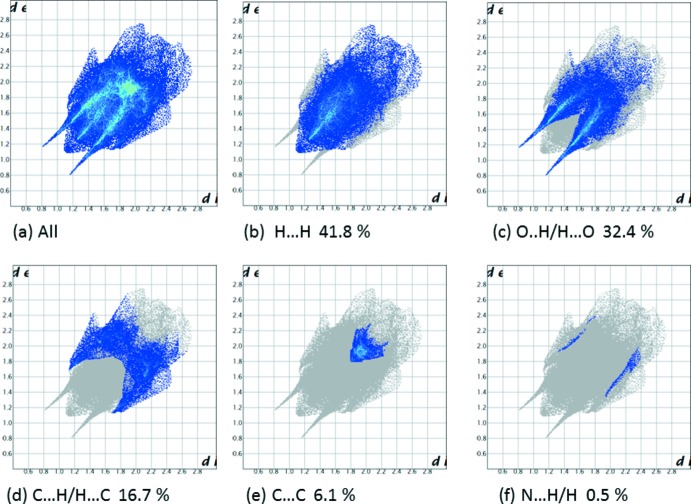
The full two-dimensional fingerprint plot for compound (II), and fingerprint plots delineated into (*b*) H⋯H, (*c*) O⋯H/H⋯O, (*d*) C⋯·H/H⋯C, (*e*) C⋯C and (*f*) N⋯H/H⋯N contacts.

The fingerprint plots are given in Figs. 8 ▸ and 9 ▸. For compound (I), they reveal that the principal inter­molecular contacts are H⋯H at 41.3% (Fig. 8 ▸ *b*) and O⋯H/H⋯O at 35.3% (Fig. 8 ▸ *c*), followed by the C⋯H/H⋯C contacts at 11.8% (Fig. 8 ▸ *d*). For compound (II), they reveal a similar trend, with the principal inter­molecular contacts being H⋯H at 41.8% (Fig. 9 ▸ *b*) and O⋯H/H⋯O at 32.4% (Fig. 9 ▸ *c*), followed by the C⋯H/H⋯C contacts at 16.7% (Fig. 9 ▸ *d*). In both compounds, the H⋯H inter­molecular contacts predominate, followed by the O⋯H/H⋯O contacts. However the C⋯H/H⋯C contacts are significantly different 11.8% *cf*. 16.7% for (I) and (II), respectively.

## Database survey   

A search of the Cambridge Structural Database (CSD, V5.40, update February 2019; Groom *et al.*, 2016 ▸) for 2-[(2-oxo-2*H*-chromen-4-yl)­oxy]acetamide derivatives gave two hits. They include 2-[(2-oxo-2*H*-chromen-4-yl)­oxy]-*N*-(1-phenyl­eth­yl)acetamide (CSD refcode PUWMEB; Govindhan, Subramanian, Chennakesava Rao *et al.*, 2015 ▸) and *N*-(3,5-di­methyl­adamantan-1-yl)-2-[(2-oxo-2*H*-chromen-4-yl)­oxy]prop­an­a­mide (SEFRAY; Rambabu *et al.*, 2012 ▸).

A search for linear and angular pyran­ocoumarin (psoralene class) structures gave 35 hits. They include four reports, CSD refcodes AMYROL [Kato, 1970 ▸: seselin (smyrolin)]; AMYROL01 [Bauri *et al.*, 2006 ▸; seselin (redetermination)]; FUGVOS [Thailambal & Pattabhi, 1987 ▸: 2,3-dihy­droxy-9-hy­droxy-2(1-hy­droxy-1-methyl­eth­yl)-7*H*-furo-[3,2-*g*]-[1]-benzo­pyran-7-one; bromo­hydroxy­seselin (Bauri *et al.*, 2017*a* ▸); di­bromo­mometh­oxy­seselin (DMS) (Bauri *et al.*, 2017*b* ▸)], and a number of structures with various substituents at C3 and C4, many of which are natural products.

A CSD search found five coumarin ester structures with substituents at the 7 position (Ramasubbu *et al.*, 1982 ▸; Gnanaguru *et al.*, 1985 ▸; Parveen *et al.*, 2011 ▸; Zhuo *et al.*, 2014 ▸; Ji *et al.*, 2017 ▸). In these structures and those of *meta*-substituted coumarin esters (Abou *et al.*, 2012 ▸, 2013 ▸; Bibila Mayaya Bisseyou *et al.*, 2013 ▸; Zhang *et al.*, 2014 ▸; Gomes *et al.*, 2016 ▸; Ziki *et al.*, 2016 ▸, 2017 ▸), the pyrone rings all show three long (in the range 1.37–1.46 Å) and one short (1.32–1.34 Å) C—C distances, suggesting that the electronic density is preferentially located in the short C—C bond at the pyrone ring. This pattern is clearly repeated here with C1—C6 = 1.3883 (18) and 1.394 (11) Å, C6—C7 = 1.4538 (15) and 1.398 (12) Å, C7—C8 = 1.3444 (17) and 1.352 (12) Å and C8—C9 = 1.4338 (18) and 1.433 (12) Å.

Intra­molecular C—H⋯O short contacts similar to that observed in the title compounds were found in five compounds in the CSD: LISLAB, 1-(1-pyrrolidinylcarbon­yl)cyclo­propyl sulfamate (Morin *et al.*, 2007 ▸), PEQHAU, 2-[30-(400-chloro­phen­yl)-40,60-di­meth­oxy­indol-70-yl]glyoxyl-1-pyrrolidine (Black *et al.*, 1997 ▸), QIBBEJ, [2-hy­droxy-5-(2-hy­droxy­benzo­yl)phen­yl](pyrrolidin-1-yl)-methanone (Holtz *et al.*, 2007 ▸), SINHAZ, 2-meth­oxy-1-(1-pyrrolidinylcarbon­yl)naphthalene (Sakamoto *et al.*, 2007 ▸) and TAJDIR, (4S,5S)-4,5-bispyrrolidinylcarbon­yl)-2,2-dimethyl-1,3-dioxolane (Garcia *et al.*, 1991 ▸).

## Synthesis and crystallization   

**Compound (I)** To a solution of 1 equiv. of 4-(2-(piperazine-1-yl)eth­oxy)-2*H*-chromen-2-one (1.0 g) in di­chloro­methane (10 ml) at 273–278 K were added tri­ethyl­amine (0.7 g, 2.0 equiv.) followed by iodo­acetamide (1.0 g, 0.5 equiv.), and the reaction mixture was stirred at the same temperature for 1 h. On completion of the reaction (monitored by TLC), the reaction mixture was diluted with di­chloro­methane and water (10 ml). The organic layer was separated and washed with brine solution. It was then dried over anhydrous sodium sulfate, filtered and then evaporated under reduced pressure giving compound (I) as a white solid, which was then washed with hexane and dried under vacuum. Colourless block-like crystals of compound (I) were obtained by slow evaporation of a solution in chloro­form (4 ml) and methanol (1 ml).

**Compound (II)** *N*,*N*-Diiso­propyl­ethyl­amine (DIPEA; 1.82 g, 3.1 equiv.) was added to a mixture of 2-(2-oxo-2*H*-chromen-4-yl­oxy)acetic acid (1.0 g, 1.0 equiv.), 1-ethyl-3-(3-di­methyl­amino­prop­yl)carbodi­imide (EDCI; 1.0 g, 1.2 equiv.), 1-hy­droxy­benzotriazole hydrate (HOBt; 0.61 g, 1.0 equiv.), 2,4-di­meth­oxy­benzyl­amine (0.8 g, 1.0 equiv.) in *N,N*-di­methyl­formamide (5 ml) at 273–278 K. The temperature of the mixture was raised to ambient temperature and stirred for 8 h. Progress of the reaction was monitored by TLC (mobile phase: ethyl acetate/hexa­ne). After completion of the reaction, the mixture was poured into ice–water and compound (II) was obtained as a white solid. It was then filtered, washed with hexane and dried under vacuum. Colourless block-like crystals of compound (II) were obtained by slow evaporation of a solution in chloro­form (5 ml).

## Refinement   

Crystal data, data collection and structure refinement details are summarized in Table 3 ▸. For both compounds the H atoms were positioned geometrically and constrained to ride on their parent atoms: N—H = 0.86 Å and C–H = 0.93—0.97Å with *U*_iso_(H) = 1.5*U*_eq_(C-meth­yl) and 1.2*U*_eq_(N, C) for other H atoms.

## Supplementary Material

Crystal structure: contains datablock(s) global, I, II. DOI: 10.1107/S2056989019003736/su5482sup1.cif

Structure factors: contains datablock(s) I. DOI: 10.1107/S2056989019003736/su5482Isup2.hkl

Structure factors: contains datablock(s) II. DOI: 10.1107/S2056989019003736/su5482IIsup3.hkl

Supporting information file. DOI: 10.1107/S2056989019003736/su5482Isup4.cml

Supporting information file. DOI: 10.1107/S2056989019003736/su5482IIsup5.cml

CCDC references: 1891497, 1891496

Additional supporting information:  crystallographic information; 3D view; checkCIF report

## Figures and Tables

**Figure 2:**
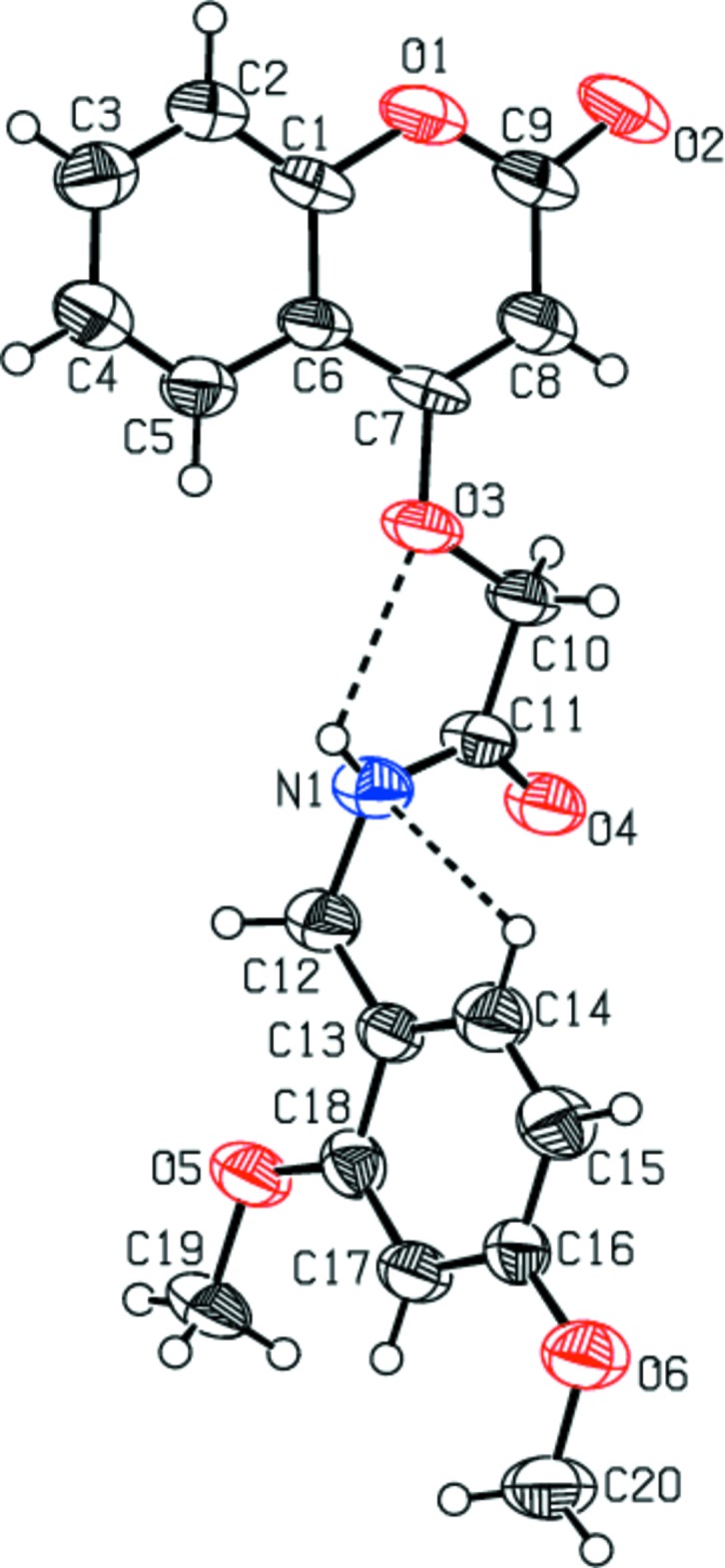
The mol­ecular structure of compound (II), with the atom labelling. Displacement ellipsoids are drawn at the 50% probability level. Intra­molecular contacts (Table 2 ▸) are shown as dashed lines.

**Table 1:** Hydrogen-bond geometry (Å, °) for (I) *Cg*1 is the centroid of the C1–C6 ring.

*D*—H⋯*A*	*D*—H	H⋯*A*	*D*⋯*A*	*D*—H⋯*A*
N3—H3*B*⋯N2	0.86	2.41	2.7716 (15)	106
C12—H12*A*⋯O4	0.97	2.35	2.7473 (15)	104
N3—H3*A*⋯O4^i^	0.86	2.05	2.8886 (15)	166
C8—H8⋯O2^ii^	0.93	2.56	3.3953 (16)	150
C10—H10*A*⋯O5^iii^	0.97	2.49	3.4506 (18)	173
C10—H10*B*⋯O2^ii^	0.97	2.42	3.3346 (16)	157
C14—H14*A*⋯O2^ii^	0.97	2.54	3.4012 (17)	148
C14—H14*B*⋯*Cg*1^i^	0.97	2.80	3.614 (2)	142

Symmetry codes: (i) 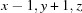; (ii) 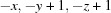; (iii) 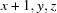.

**Table 2:** Hydrogen-bond geometry (Å, °) for (II)

*D*—H⋯*A*	*D*—H	H⋯*A*	*D*⋯*A*	*D*—H⋯*A*
N1—H1⋯O3	0.86	2.31	2.669 (2)	105
C14—H14⋯N1	0.93	2.59	2.923 (2)	101
N1—H1⋯O4^i^	0.86	2.09	2.900 (2)	156
C3—H3⋯O5^ii^	0.93	2.49	3.419 (2)	175
C5—H5⋯O4^i^	0.93	2.43	3.307 (2)	157
C15—H15⋯O4^iii^	0.93	2.51	3.399 (2)	160

Symmetry codes: (i) 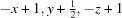; (ii) 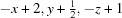; (iii) 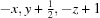.

**Table 3:** Experimental details

	(I)	(II)
Crystal data		
Chemical formula	C_17_H_19_N_3_O_5_	C_20_H_19_NO_6_
*M*_r_	345.35	369.36
Crystal system, space group	Triclinic, *P* 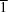	Monoclinic, *P*2_1_
Temperature (K)	293	296
*a*, *b*, *c* (Å)	8.5260 (3), 8.8415 (3), 11.9462 (4)	7.2779 (2), 8.5759 (3), 14.4099 (5)
α, β, γ (°)	88.660 (2), 69.568 (2), 70.724 (2)	90, 93.796 (5), 90
*V* (Å^3^)	792.27 (5)	897.41 (5)
*Z*	2	2
Radiation type	Mo *K*α	Mo *K*α
μ (mm^−1^)	0.11	0.10
Crystal size (mm)	0.25 × 0.24 × 0.20	0.30 × 0.25 × 0.20

Data collection		
Diffractometer	Bruker Kappa APEXII CCD	Bruker Kappa APEXII CCD
Absorption correction	Multi-scan (*SADABS*; Bruker, 2008 ▸)	Multi-scan (*SADABS*; Bruker, 2008 ▸)
*T*_min_, *T*_max_	0.756, 0.824	0.763, 0.852
No. of measured, independent and observed [*I* > 2σ(*I*)] reflections	12022, 3382, 2947	4058, 2630, 1623
*R*_int_	0.027	0.088
(sin θ/λ)_max_ (Å^−1^)	0.637	0.595

Refinement		
*R*[*F* ^2^ > 2σ(*F* ^2^)], *wR*(*F* ^2^), *S*	0.039, 0.110, 1.04	0.083, 0.243, 0.98
No. of reflections	3382	2630
No. of parameters	227	247
No. of restraints	0	1
H-atom treatment	H-atom parameters constrained	H-atom parameters constrained
Δρ_max_, Δρ_min_ (e Å^−3^)	0.30, −0.18	0.28, −0.29

Computer programs: *APEX2* and *SAINT* (Bruker, 2008 ▸), *SHELXS2018* (Sheldrick, 2008 ▸), *SHELXL2018* (Sheldrick, 2015 ▸), *ORTEP-3 for Windows* and *WinGX* (Farrugia, 2012 ▸), *Mercury* (Macrae *et al.*, 2008 ▸), *publCIF* (Westrip, 2010 ▸) and *PLATON* (Spek, 2009 ▸).

## supplementary crystallographic information

### 2-(4-{2-[(2-Oxo-2H-chromen-4-yl)oxy]acetyl}piperazin-1-yl)acetamide (I) Crystal data

**Table:**

C_17_H_19_N_3_O_5_	*Z* = 2
*M**_r_* = 345.35	*F*(000) = 364
Triclinic, *P*1	*D*_x_ = 1.448 Mg m^−^^3^
*a* = 8.5260 (3) Å	Mo *K*α radiation, λ = 0.71073 Å
*b* = 8.8415 (3) Å	Cell parameters from 3382 reflections
*c* = 11.9462 (4) Å	θ = 1.8–26.9°
α = 88.660 (2)°	µ = 0.11 mm^−^^1^
β = 69.568 (2)°	*T* = 293 K
γ = 70.724 (2)°	Block, colourless
*V* = 792.27 (5) Å^3^	0.25 × 0.24 × 0.20 mm

### 2-(4-{2-[(2-Oxo-2H-chromen-4-yl)oxy]acetyl}piperazin-1-yl)acetamide (I) Data collection

**Table:**

Bruker Kappa APEXII CCD diffractometer	2947 reflections with *I* > 2σ(*I*)
ω and φ scans	*R*_int_ = 0.027
Absorption correction: multi-scan (*SADABS*; Bruker, 2008)	θ_max_ = 26.9°, θ_min_ = 1.8°
*T*_min_ = 0.756, *T*_max_ = 0.824	*h* = −10→10
12022 measured reflections	*k* = −11→11
3382 independent reflections	*l* = −15→15

### 2-(4-{2-[(2-Oxo-2H-chromen-4-yl)oxy]acetyl}piperazin-1-yl)acetamide (I) Refinement

**Table:**

Refinement on *F*^2^	Secondary atom site location: difference Fourier map
Least-squares matrix: full	Hydrogen site location: inferred from neighbouring sites
*R*[*F*^2^ > 2σ(*F*^2^)] = 0.039	H-atom parameters constrained
*wR*(*F*^2^) = 0.110	*w* = 1/[σ^2^(*F*_o_^2^) + (0.0556*P*)^2^ + 0.2007*P*] where *P* = (*F*_o_^2^ + 2*F*_c_^2^)/3
*S* = 1.04	(Δ/σ)_max_ < 0.001
3382 reflections	Δρ_max_ = 0.30 e Å^−^^3^
227 parameters	Δρ_min_ = −0.18 e Å^−^^3^
0 restraints	Extinction correction: (SHELXL2018; Sheldrick, 2015), Fc^*^=kFc[1+0.001xFc^2^λ^3^/sin(2θ)]^-1/4^
Primary atom site location: structure-invariant direct methods	Extinction coefficient: 0.020 (4)

### 2-(4-{2-[(2-Oxo-2H-chromen-4-yl)oxy]acetyl}piperazin-1-yl)acetamide (I) Special details

**Table:**

Geometry. All esds (except the esd in the dihedral angle between two l.s. planes) are estimated using the full covariance matrix. The cell esds are taken into account individually in the estimation of esds in distances, angles and torsion angles; correlations between esds in cell parameters are only used when they are defined by crystal symmetry. An approximate (isotropic) treatment of cell esds is used for estimating esds involving l.s. planes.

### 2-(4-{2-[(2-Oxo-2H-chromen-4-yl)oxy]acetyl}piperazin-1-yl)acetamide (I) Fractional atomic coordinates and isotropic or equivalent isotropic displacement parameters (Å^2^)

**Table:**

	*x*	*y*	*z*	*U*_iso_*/*U*_eq_	
C1	0.57138 (16)	0.24849 (15)	0.44898 (11)	0.0326 (3)	
C2	0.72086 (18)	0.15454 (16)	0.47279 (13)	0.0396 (3)	
H2	0.713439	0.076848	0.526334	0.047*	
C3	0.87983 (18)	0.17916 (17)	0.41538 (13)	0.0426 (3)	
H3	0.980829	0.116794	0.430161	0.051*	
C4	0.89217 (18)	0.29534 (18)	0.33584 (13)	0.0419 (3)	
H4	1.000730	0.310220	0.297651	0.050*	
C5	0.74271 (16)	0.38890 (16)	0.31349 (12)	0.0355 (3)	
H5	0.750512	0.467720	0.260957	0.043*	
C6	0.57975 (15)	0.36540 (14)	0.36965 (11)	0.0297 (3)	
C7	0.41622 (16)	0.45736 (14)	0.35108 (11)	0.0298 (3)	
C8	0.26528 (16)	0.42648 (15)	0.40841 (12)	0.0359 (3)	
H8	0.162437	0.483635	0.393790	0.043*	
C9	0.25981 (17)	0.30714 (16)	0.49166 (13)	0.0385 (3)	
C10	0.28057 (16)	0.68340 (15)	0.26066 (12)	0.0328 (3)	
H10A	0.295441	0.787474	0.249025	0.039*	
H10B	0.176835	0.696178	0.332561	0.039*	
C11	0.25178 (15)	0.62523 (14)	0.15331 (11)	0.0316 (3)	
C12	0.11451 (17)	0.69477 (15)	0.00279 (11)	0.0342 (3)	
H12A	0.191175	0.585566	−0.031236	0.041*	
H12B	0.137190	0.766053	−0.059147	0.041*	
C13	−0.07778 (17)	0.70594 (14)	0.04377 (13)	0.0358 (3)	
H13A	−0.104008	0.680104	−0.024677	0.043*	
H13B	−0.097577	0.627835	0.100571	0.043*	
C14	−0.15445 (17)	0.91151 (15)	0.20136 (11)	0.0349 (3)	
H14A	−0.176974	0.839746	0.262958	0.042*	
H14B	−0.231186	1.020451	0.236057	0.042*	
C15	0.03821 (17)	0.90093 (14)	0.16159 (12)	0.0348 (3)	
H15A	0.058654	0.980127	0.105913	0.042*	
H15B	0.064622	0.924109	0.230622	0.042*	
C16	−0.38171 (17)	0.87031 (15)	0.14408 (15)	0.0423 (3)	
H16A	−0.401742	0.814610	0.215885	0.051*	
H16B	−0.396724	0.810638	0.083757	0.051*	
C17	−0.52246 (17)	1.03707 (16)	0.17290 (14)	0.0419 (3)	
N1	0.15528 (13)	0.73936 (12)	0.10337 (9)	0.0319 (2)	
N2	−0.19807 (13)	0.86796 (11)	0.10060 (9)	0.0308 (2)	
N3	−0.47248 (16)	1.15212 (14)	0.11445 (12)	0.0465 (3)	
H3A	−0.547778	1.248448	0.125737	0.056*	
H3B	−0.364686	1.130611	0.065147	0.056*	
O1	0.41560 (12)	0.22028 (11)	0.50832 (9)	0.0416 (2)	
O2	0.12892 (14)	0.27595 (14)	0.55044 (11)	0.0566 (3)	
O3	0.43529 (11)	0.57057 (11)	0.27551 (9)	0.0382 (2)	
O4	0.31673 (15)	0.48319 (11)	0.11304 (10)	0.0529 (3)	
O5	−0.67279 (15)	1.05727 (14)	0.24444 (14)	0.0753 (4)	

### 2-(4-{2-[(2-Oxo-2H-chromen-4-yl)oxy]acetyl}piperazin-1-yl)acetamide (I) Atomic displacement parameters (Å^2^)

**Table:**

	*U*^11^	*U*^22^	*U*^33^	*U*^12^	*U*^13^	*U*^23^
C1	0.0309 (6)	0.0317 (6)	0.0322 (6)	−0.0043 (5)	−0.0139 (5)	0.0023 (5)
C2	0.0411 (7)	0.0344 (6)	0.0401 (7)	−0.0007 (5)	−0.0225 (6)	0.0044 (5)
C3	0.0347 (7)	0.0414 (7)	0.0480 (8)	0.0031 (5)	−0.0252 (6)	−0.0034 (6)
C4	0.0283 (6)	0.0502 (8)	0.0437 (8)	−0.0067 (6)	−0.0150 (6)	−0.0027 (6)
C5	0.0309 (6)	0.0402 (7)	0.0330 (7)	−0.0080 (5)	−0.0128 (5)	0.0042 (5)
C6	0.0275 (6)	0.0309 (6)	0.0280 (6)	−0.0032 (5)	−0.0132 (5)	0.0006 (5)
C7	0.0298 (6)	0.0305 (6)	0.0285 (6)	−0.0061 (5)	−0.0141 (5)	0.0057 (5)
C8	0.0282 (6)	0.0379 (7)	0.0415 (7)	−0.0068 (5)	−0.0173 (5)	0.0110 (5)
C9	0.0312 (6)	0.0395 (7)	0.0431 (7)	−0.0089 (5)	−0.0146 (5)	0.0109 (6)
C10	0.0287 (6)	0.0318 (6)	0.0394 (7)	−0.0072 (5)	−0.0177 (5)	0.0130 (5)
C11	0.0277 (6)	0.0270 (6)	0.0371 (7)	−0.0049 (4)	−0.0129 (5)	0.0088 (5)
C12	0.0354 (6)	0.0293 (6)	0.0328 (6)	−0.0015 (5)	−0.0153 (5)	0.0001 (5)
C13	0.0385 (7)	0.0252 (6)	0.0436 (7)	−0.0057 (5)	−0.0195 (6)	−0.0003 (5)
C14	0.0347 (6)	0.0295 (6)	0.0336 (7)	−0.0003 (5)	−0.0142 (5)	0.0009 (5)
C15	0.0372 (7)	0.0236 (5)	0.0443 (7)	−0.0008 (5)	−0.0243 (6)	−0.0003 (5)
C16	0.0325 (7)	0.0316 (6)	0.0635 (9)	−0.0104 (5)	−0.0192 (6)	0.0099 (6)
C17	0.0281 (6)	0.0363 (7)	0.0591 (9)	−0.0070 (5)	−0.0171 (6)	0.0049 (6)
N1	0.0322 (5)	0.0250 (5)	0.0363 (6)	−0.0012 (4)	−0.0179 (4)	0.0017 (4)
N2	0.0278 (5)	0.0250 (5)	0.0388 (6)	−0.0047 (4)	−0.0153 (4)	0.0032 (4)
N3	0.0340 (6)	0.0324 (6)	0.0650 (8)	−0.0031 (5)	−0.0166 (6)	0.0110 (5)
O1	0.0351 (5)	0.0414 (5)	0.0469 (6)	−0.0093 (4)	−0.0178 (4)	0.0197 (4)
O2	0.0375 (5)	0.0654 (7)	0.0673 (7)	−0.0209 (5)	−0.0181 (5)	0.0328 (6)
O3	0.0280 (4)	0.0439 (5)	0.0448 (5)	−0.0100 (4)	−0.0190 (4)	0.0205 (4)
O4	0.0659 (7)	0.0272 (5)	0.0597 (7)	0.0043 (4)	−0.0346 (6)	0.0017 (4)
O5	0.0340 (6)	0.0528 (7)	0.1128 (11)	−0.0086 (5)	−0.0018 (6)	0.0128 (7)

### 2-(4-{2-[(2-Oxo-2H-chromen-4-yl)oxy]acetyl}piperazin-1-yl)acetamide (I) Geometric parameters (Å, º)

**Table:**

C1—O1	1.3718 (16)	C11—N1	1.3482 (15)
C1—C6	1.3883 (18)	C12—N1	1.4574 (16)
C1—C2	1.3922 (17)	C12—C13	1.5073 (18)
C2—C3	1.376 (2)	C12—H12A	0.9700
C2—H2	0.9300	C12—H12B	0.9700
C3—C4	1.387 (2)	C13—N2	1.4677 (15)
C3—H3	0.9300	C13—H13A	0.9700
C4—C5	1.3823 (18)	C13—H13B	0.9700
C4—H4	0.9300	C14—N2	1.4712 (16)
C5—C6	1.3990 (17)	C14—C15	1.5127 (18)
C5—H5	0.9300	C14—H14A	0.9700
C6—C7	1.4538 (15)	C14—H14B	0.9700
C7—O3	1.3439 (15)	C15—N1	1.4632 (15)
C7—C8	1.3444 (17)	C15—H15A	0.9700
C8—C9	1.4338 (18)	C15—H15B	0.9700
C8—H8	0.9300	C16—N2	1.4604 (16)
C9—O2	1.2089 (16)	C16—C17	1.5164 (18)
C9—O1	1.3774 (15)	C16—H16A	0.9700
C10—O3	1.4328 (13)	C16—H16B	0.9700
C10—C11	1.5177 (18)	C17—O5	1.2232 (18)
C10—H10A	0.9700	C17—N3	1.3226 (19)
C10—H10B	0.9700	N3—H3A	0.8600
C11—O4	1.2241 (15)	N3—H3B	0.8600
			
O1—C1—C6	121.80 (11)	C13—C12—H12B	109.6
O1—C1—C2	116.65 (12)	H12A—C12—H12B	108.1
C6—C1—C2	121.54 (12)	N2—C13—C12	111.22 (10)
C3—C2—C1	118.55 (13)	N2—C13—H13A	109.4
C3—C2—H2	120.7	C12—C13—H13A	109.4
C1—C2—H2	120.7	N2—C13—H13B	109.4
C2—C3—C4	121.20 (12)	C12—C13—H13B	109.4
C2—C3—H3	119.4	H13A—C13—H13B	108.0
C4—C3—H3	119.4	N2—C14—C15	111.67 (10)
C5—C4—C3	119.83 (13)	N2—C14—H14A	109.3
C5—C4—H4	120.1	C15—C14—H14A	109.3
C3—C4—H4	120.1	N2—C14—H14B	109.3
C4—C5—C6	120.20 (13)	C15—C14—H14B	109.3
C4—C5—H5	119.9	H14A—C14—H14B	107.9
C6—C5—H5	119.9	N1—C15—C14	109.84 (10)
C1—C6—C5	118.66 (11)	N1—C15—H15A	109.7
C1—C6—C7	117.34 (11)	C14—C15—H15A	109.7
C5—C6—C7	123.99 (11)	N1—C15—H15B	109.7
O3—C7—C8	126.49 (11)	C14—C15—H15B	109.7
O3—C7—C6	113.36 (10)	H15A—C15—H15B	108.2
C8—C7—C6	120.14 (11)	N2—C16—C17	114.84 (10)
C7—C8—C9	121.26 (11)	N2—C16—H16A	108.6
C7—C8—H8	119.4	C17—C16—H16A	108.6
C9—C8—H8	119.4	N2—C16—H16B	108.6
O2—C9—O1	116.18 (12)	C17—C16—H16B	108.6
O2—C9—C8	125.71 (12)	H16A—C16—H16B	107.5
O1—C9—C8	118.11 (11)	O5—C17—N3	124.21 (13)
O3—C10—C11	110.44 (10)	O5—C17—C16	119.73 (13)
O3—C10—H10A	109.6	N3—C17—C16	116.04 (12)
C11—C10—H10A	109.6	C11—N1—C12	120.29 (10)
O3—C10—H10B	109.6	C11—N1—C15	125.01 (11)
C11—C10—H10B	109.6	C12—N1—C15	111.98 (9)
H10A—C10—H10B	108.1	C16—N2—C13	109.10 (10)
O4—C11—N1	122.28 (12)	C16—N2—C14	109.88 (10)
O4—C11—C10	121.53 (11)	C13—N2—C14	109.94 (9)
N1—C11—C10	116.17 (10)	C17—N3—H3A	120.0
N1—C12—C13	110.40 (10)	C17—N3—H3B	120.0
N1—C12—H12A	109.6	H3A—N3—H3B	120.0
C13—C12—H12A	109.6	C1—O1—C9	121.30 (10)
N1—C12—H12B	109.6	C7—O3—C10	119.29 (9)
			
O1—C1—C2—C3	−179.85 (11)	N2—C16—C17—O5	−155.82 (15)
C6—C1—C2—C3	−0.22 (19)	N2—C16—C17—N3	25.9 (2)
C1—C2—C3—C4	0.4 (2)	O4—C11—N1—C12	4.18 (19)
C2—C3—C4—C5	0.2 (2)	C10—C11—N1—C12	−177.62 (10)
C3—C4—C5—C6	−0.8 (2)	O4—C11—N1—C15	163.96 (13)
O1—C1—C6—C5	179.20 (11)	C10—C11—N1—C15	−17.84 (18)
C2—C1—C6—C5	−0.42 (18)	C13—C12—N1—C11	105.42 (13)
O1—C1—C6—C7	−0.25 (18)	C13—C12—N1—C15	−56.81 (14)
C2—C1—C6—C7	−179.87 (11)	C14—C15—N1—C11	−105.06 (14)
C4—C5—C6—C1	0.93 (19)	C14—C15—N1—C12	56.17 (14)
C4—C5—C6—C7	−179.66 (12)	C17—C16—N2—C13	−163.63 (12)
C1—C6—C7—O3	178.34 (10)	C17—C16—N2—C14	75.77 (15)
C5—C6—C7—O3	−1.09 (17)	C12—C13—N2—C16	−177.13 (11)
C1—C6—C7—C8	−0.84 (18)	C12—C13—N2—C14	−56.57 (14)
C5—C6—C7—C8	179.74 (12)	C15—C14—N2—C16	176.60 (10)
O3—C7—C8—C9	−176.93 (12)	C15—C14—N2—C13	56.50 (13)
C6—C7—C8—C9	2.1 (2)	C6—C1—O1—C9	0.03 (18)
C7—C8—C9—O2	176.98 (14)	C2—C1—O1—C9	179.66 (12)
C7—C8—C9—O1	−2.3 (2)	O2—C9—O1—C1	−178.15 (12)
O3—C10—C11—O4	21.02 (17)	C8—C9—O1—C1	1.20 (19)
O3—C10—C11—N1	−157.19 (10)	C8—C7—O3—C10	6.90 (19)
N1—C12—C13—N2	56.80 (14)	C6—C7—O3—C10	−172.21 (10)
N2—C14—C15—N1	−56.02 (13)	C11—C10—O3—C7	−94.52 (13)

### 2-(4-{2-[(2-Oxo-2H-chromen-4-yl)oxy]acetyl}piperazin-1-yl)acetamide (I) Hydrogen-bond geometry (Å, º)

*Cg*1 is the centroid of the C1–C6 ring.

**Table:**

*D*—H···*A*	*D*—H	H···*A*	*D*···*A*	*D*—H···*A*
N3—H3*B*···N2	0.86	2.41	2.7716 (15)	106
C12—H12*A*···O4	0.97	2.35	2.7473 (15)	104
N3—H3*A*···O4^i^	0.86	2.05	2.8886 (15)	166
C8—H8···O2^ii^	0.93	2.56	3.3953 (16)	150
C10—H10*A*···O5^iii^	0.97	2.49	3.4506 (18)	173
C10—H10*B*···O2^ii^	0.97	2.42	3.3346 (16)	157
C14—H14*A*···O2^ii^	0.97	2.54	3.4012 (17)	148
C14—H14*B*···*Cg*1^i^	0.97	2.80	3.614 (2)	142

Symmetry codes: (i) *x*−1, *y*+1, *z*; (ii) −*x*, −*y*+1, −*z*+1; (iii) *x*+1, *y*, *z*.

### N-(2,4-Dimethoxybenzyl)-2-[(2-oxo-2H-chromen-4-yl)oxy]acetamide (II) Crystal data

**Table:**

C_20_H_19_NO_6_	*F*(000) = 388
*M**_r_* = 369.36	*D*_x_ = 1.367 Mg m^−^^3^
Monoclinic, *P*2_1_	Mo *K*α radiation, λ = 0.71073 Å
*a* = 7.2779 (2) Å	Cell parameters from 2630 reflections
*b* = 8.5759 (3) Å	θ = 1.4–25.0°
*c* = 14.4099 (5) Å	µ = 0.10 mm^−^^1^
β = 93.796 (5)°	*T* = 296 K
*V* = 897.41 (5) Å^3^	Block, colourless
*Z* = 2	0.30 × 0.25 × 0.20 mm

### N-(2,4-Dimethoxybenzyl)-2-[(2-oxo-2H-chromen-4-yl)oxy]acetamide (II) Data collection

**Table:**

Bruker Kappa APEXII CCD diffractometer	1623 reflections with *I* > 2σ(*I*)
ω and φ scans	*R*_int_ = 0.088
Absorption correction: multi-scan (*SADABS*; Bruker, 2008)	θ_max_ = 25.0°, θ_min_ = 1.4°
*T*_min_ = 0.763, *T*_max_ = 0.852	*h* = −8→8
4058 measured reflections	*k* = −9→9
2630 independent reflections	*l* = −17→16

### N-(2,4-Dimethoxybenzyl)-2-[(2-oxo-2H-chromen-4-yl)oxy]acetamide (II) Refinement

**Table:**

Refinement on *F*^2^	Secondary atom site location: difference Fourier map
Least-squares matrix: full	Hydrogen site location: inferred from neighbouring sites
*R*[*F*^2^ > 2σ(*F*^2^)] = 0.083	H-atom parameters constrained
*wR*(*F*^2^) = 0.243	*w* = 1/[σ^2^(*F*_o_^2^) + (0.1336*P*)^2^] where *P* = (*F*_o_^2^ + 2*F*_c_^2^)/3
*S* = 0.98	(Δ/σ)_max_ < 0.001
2630 reflections	Δρ_max_ = 0.28 e Å^−^^3^
247 parameters	Δρ_min_ = −0.29 e Å^−^^3^
1 restraint	Extinction correction: (SHELXL2018; Sheldrick, 2015), Fc^*^=kFc[1+0.001xFc^2^λ^3^/sin(2θ)]^-1/4^
Primary atom site location: structure-invariant direct methods	Extinction coefficient: 0.042 (15)

### N-(2,4-Dimethoxybenzyl)-2-[(2-oxo-2H-chromen-4-yl)oxy]acetamide (II) Special details

**Table:**

Geometry. All esds (except the esd in the dihedral angle between two l.s. planes) are estimated using the full covariance matrix. The cell esds are taken into account individually in the estimation of esds in distances, angles and torsion angles; correlations between esds in cell parameters are only used when they are defined by crystal symmetry. An approximate (isotropic) treatment of cell esds is used for estimating esds involving l.s. planes.

### N-(2,4-Dimethoxybenzyl)-2-[(2-oxo-2H-chromen-4-yl)oxy]acetamide (II) Fractional atomic coordinates and isotropic or equivalent isotropic displacement parameters (Å^2^)

**Table:**

	*x*	*y*	*z*	*U*_iso_*/*U*_eq_	
C9	0.7608 (13)	0.3941 (14)	0.8943 (6)	0.063 (3)	
C1	0.9935 (12)	0.5490 (11)	0.8258 (6)	0.053 (2)	
C2	1.1588 (13)	0.6195 (14)	0.8397 (7)	0.064 (3)	
H2	1.225084	0.612966	0.897037	0.077*	
C3	1.2277 (13)	0.7016 (13)	0.7673 (7)	0.067 (3)	
H3	1.340590	0.752129	0.775680	0.080*	
C4	1.1281 (13)	0.7085 (14)	0.6818 (7)	0.065 (3)	
H4	1.175985	0.762353	0.632809	0.078*	
C5	0.9635 (12)	0.6383 (12)	0.6689 (6)	0.056 (3)	
H5	0.898157	0.645409	0.611398	0.067*	
C6	0.8876 (11)	0.5536 (11)	0.7417 (6)	0.048 (2)	
C7	0.7177 (12)	0.4769 (10)	0.7354 (5)	0.047 (2)	
C8	0.6529 (13)	0.3977 (13)	0.8075 (7)	0.060 (3)	
H8	0.540283	0.346313	0.800782	0.072*	
C10	0.4698 (11)	0.3848 (12)	0.6345 (6)	0.052 (2)	
H10A	0.365667	0.432162	0.662342	0.062*	
H10B	0.493567	0.284583	0.664029	0.062*	
C11	0.4238 (11)	0.3613 (11)	0.5313 (6)	0.046 (2)	
C12	0.4617 (11)	0.4368 (12)	0.3725 (6)	0.053 (2)	
H12A	0.461193	0.326030	0.358746	0.064*	
H12B	0.563467	0.482854	0.341984	0.064*	
C13	0.2869 (10)	0.5056 (10)	0.3312 (5)	0.043 (2)	
C14	0.1620 (12)	0.5896 (12)	0.3804 (7)	0.059 (3)	
H14	0.190916	0.609186	0.443136	0.071*	
C15	−0.0013 (12)	0.6448 (14)	0.3410 (7)	0.062 (3)	
H15	−0.081969	0.699614	0.376388	0.074*	
C16	−0.0442 (11)	0.6175 (12)	0.2473 (6)	0.052 (2)	
C17	0.0765 (11)	0.5380 (11)	0.1954 (6)	0.052 (2)	
H17	0.048794	0.523211	0.132044	0.062*	
C18	0.2389 (11)	0.4798 (11)	0.2368 (6)	0.048 (2)	
C19	0.3259 (14)	0.3671 (17)	0.0943 (6)	0.080 (4)	
H19A	0.212442	0.310013	0.086580	0.120*	
H19B	0.423474	0.306460	0.070819	0.120*	
H19C	0.313972	0.463616	0.060635	0.120*	
C20	−0.2598 (13)	0.6447 (17)	0.1157 (7)	0.085 (4)	
H20A	−0.162895	0.679847	0.078717	0.127*	
H20B	−0.371543	0.699158	0.097180	0.127*	
H20C	−0.278034	0.534745	0.106668	0.127*	
N1	0.4962 (10)	0.4564 (9)	0.4721 (5)	0.051 (2)	
H1	0.565201	0.531787	0.492909	0.061*	
O1	0.9285 (9)	0.4669 (9)	0.9009 (4)	0.068 (2)	
O2	0.7213 (10)	0.3219 (10)	0.9634 (5)	0.084 (3)	
O3	0.6246 (8)	0.4805 (7)	0.6494 (4)	0.0562 (18)	
O4	0.3165 (8)	0.2542 (8)	0.5078 (4)	0.0560 (18)	
O5	0.3671 (8)	0.3989 (9)	0.1907 (4)	0.0641 (19)	
O6	−0.2109 (8)	0.6749 (9)	0.2111 (5)	0.072 (2)	

### N-(2,4-Dimethoxybenzyl)-2-[(2-oxo-2H-chromen-4-yl)oxy]acetamide (II) Atomic displacement parameters (Å^2^)

**Table:**

	*U*^11^	*U*^22^	*U*^33^	*U*^12^	*U*^13^	*U*^23^
C9	0.079 (6)	0.082 (8)	0.028 (5)	−0.001 (6)	−0.004 (4)	−0.001 (5)
C1	0.074 (6)	0.060 (6)	0.025 (5)	0.007 (5)	−0.004 (4)	0.000 (4)
C2	0.065 (6)	0.087 (9)	0.039 (5)	−0.006 (6)	−0.009 (4)	0.004 (6)
C3	0.063 (6)	0.079 (8)	0.056 (7)	−0.010 (6)	−0.011 (5)	0.002 (6)
C4	0.073 (6)	0.080 (8)	0.042 (6)	−0.006 (6)	0.002 (4)	0.007 (6)
C5	0.057 (5)	0.066 (7)	0.042 (5)	−0.008 (5)	−0.008 (4)	0.009 (5)
C6	0.053 (5)	0.058 (6)	0.029 (5)	−0.002 (4)	−0.007 (4)	0.001 (4)
C7	0.071 (5)	0.049 (6)	0.018 (4)	0.002 (5)	−0.008 (4)	0.003 (4)
C8	0.068 (6)	0.075 (7)	0.036 (5)	−0.007 (5)	−0.003 (4)	0.004 (5)
C10	0.059 (5)	0.065 (7)	0.031 (5)	−0.012 (5)	−0.007 (4)	0.003 (5)
C11	0.057 (5)	0.046 (5)	0.033 (5)	−0.004 (5)	−0.007 (4)	0.004 (5)
C12	0.056 (5)	0.063 (6)	0.040 (5)	0.000 (5)	−0.002 (4)	−0.006 (5)
C13	0.049 (4)	0.048 (6)	0.032 (4)	−0.003 (4)	0.001 (3)	0.001 (4)
C14	0.059 (5)	0.072 (7)	0.048 (6)	0.006 (5)	0.000 (4)	−0.006 (5)
C15	0.063 (5)	0.077 (7)	0.046 (5)	0.012 (5)	0.007 (4)	−0.021 (6)
C16	0.043 (4)	0.073 (7)	0.041 (5)	0.011 (5)	0.005 (4)	0.001 (5)
C17	0.055 (5)	0.066 (6)	0.035 (5)	0.011 (5)	0.000 (4)	−0.001 (5)
C18	0.049 (4)	0.053 (6)	0.042 (5)	0.004 (4)	0.009 (4)	−0.003 (5)
C19	0.082 (7)	0.130 (11)	0.029 (5)	0.008 (7)	0.005 (4)	−0.006 (7)
C20	0.067 (6)	0.137 (11)	0.048 (6)	0.033 (7)	−0.012 (5)	−0.014 (7)
N1	0.065 (4)	0.046 (5)	0.041 (4)	−0.010 (4)	−0.009 (3)	0.001 (4)
O1	0.080 (4)	0.090 (5)	0.033 (4)	−0.009 (4)	−0.009 (3)	0.007 (4)
O2	0.105 (6)	0.115 (7)	0.030 (4)	−0.014 (5)	0.001 (3)	0.012 (4)
O3	0.070 (4)	0.068 (4)	0.029 (3)	−0.017 (3)	−0.012 (3)	0.007 (3)
O4	0.070 (4)	0.060 (4)	0.038 (4)	−0.012 (3)	−0.006 (3)	0.002 (3)
O5	0.067 (4)	0.090 (5)	0.035 (4)	0.015 (4)	0.002 (3)	−0.002 (4)
O6	0.065 (4)	0.102 (6)	0.048 (4)	0.026 (4)	−0.003 (3)	−0.011 (4)

### N-(2,4-Dimethoxybenzyl)-2-[(2-oxo-2H-chromen-4-yl)oxy]acetamide (II) Geometric parameters (Å, º)

**Table:**

C9—O2	1.223 (11)	C12—N1	1.451 (11)
C9—O1	1.369 (11)	C12—C13	1.490 (11)
C9—C8	1.433 (12)	C12—H12A	0.9700
C1—C2	1.350 (13)	C12—H12B	0.9700
C1—C6	1.394 (11)	C13—C14	1.391 (12)
C1—O1	1.399 (11)	C13—C18	1.400 (11)
C2—C3	1.381 (14)	C14—C15	1.367 (12)
C2—H2	0.9300	C14—H14	0.9300
C3—C4	1.389 (12)	C15—C16	1.386 (12)
C3—H3	0.9300	C15—H15	0.9300
C4—C5	1.343 (13)	C16—C17	1.372 (12)
C4—H4	0.9300	C16—O6	1.379 (10)
C5—C6	1.417 (12)	C17—C18	1.382 (11)
C5—H5	0.9300	C17—H17	0.9300
C6—C7	1.398 (12)	C18—O5	1.369 (10)
C7—C8	1.352 (12)	C19—O5	1.428 (11)
C7—O3	1.373 (9)	C19—H19A	0.9600
C8—H8	0.9300	C19—H19B	0.9600
C10—O3	1.399 (10)	C19—H19C	0.9600
C10—C11	1.516 (11)	C20—O6	1.421 (11)
C10—H10A	0.9700	C20—H20A	0.9600
C10—H10B	0.9700	C20—H20B	0.9600
C11—O4	1.239 (10)	C20—H20C	0.9600
C11—N1	1.315 (11)	N1—H1	0.8600
			
O2—C9—O1	115.5 (8)	C13—C12—H12B	108.3
O2—C9—C8	125.3 (10)	H12A—C12—H12B	107.4
O1—C9—C8	119.0 (9)	C14—C13—C18	116.6 (7)
C2—C1—C6	123.5 (9)	C14—C13—C12	124.8 (8)
C2—C1—O1	117.0 (7)	C18—C13—C12	118.5 (7)
C6—C1—O1	119.4 (8)	C15—C14—C13	123.2 (9)
C1—C2—C3	118.7 (8)	C15—C14—H14	118.4
C1—C2—H2	120.6	C13—C14—H14	118.4
C3—C2—H2	120.6	C14—C15—C16	118.7 (8)
C2—C3—C4	119.9 (9)	C14—C15—H15	120.7
C2—C3—H3	120.0	C16—C15—H15	120.7
C4—C3—H3	120.0	C17—C16—O6	123.4 (7)
C5—C4—C3	120.8 (10)	C17—C16—C15	120.3 (8)
C5—C4—H4	119.6	O6—C16—C15	116.4 (7)
C3—C4—H4	119.6	C16—C17—C18	120.3 (8)
C4—C5—C6	121.1 (8)	C16—C17—H17	119.9
C4—C5—H5	119.5	C18—C17—H17	119.9
C6—C5—H5	119.5	O5—C18—C17	124.4 (8)
C1—C6—C7	118.6 (8)	O5—C18—C13	114.6 (7)
C1—C6—C5	116.0 (8)	C17—C18—C13	120.9 (8)
C7—C6—C5	125.4 (7)	O5—C19—H19A	109.5
C8—C7—O3	121.9 (8)	O5—C19—H19B	109.5
C8—C7—C6	122.6 (7)	H19A—C19—H19B	109.5
O3—C7—C6	115.4 (7)	O5—C19—H19C	109.5
C7—C8—C9	118.9 (9)	H19A—C19—H19C	109.5
C7—C8—H8	120.6	H19B—C19—H19C	109.5
C9—C8—H8	120.6	O6—C20—H20A	109.5
O3—C10—C11	110.6 (7)	O6—C20—H20B	109.5
O3—C10—H10A	109.5	H20A—C20—H20B	109.5
C11—C10—H10A	109.5	O6—C20—H20C	109.5
O3—C10—H10B	109.5	H20A—C20—H20C	109.5
C11—C10—H10B	109.5	H20B—C20—H20C	109.5
H10A—C10—H10B	108.1	C11—N1—C12	121.3 (7)
O4—C11—N1	123.7 (7)	C11—N1—H1	119.4
O4—C11—C10	117.5 (8)	C12—N1—H1	119.4
N1—C11—C10	118.8 (8)	C9—O1—C1	121.4 (7)
N1—C12—C13	115.9 (8)	C7—O3—C10	118.0 (6)
N1—C12—H12A	108.3	C18—O5—C19	117.5 (7)
C13—C12—H12A	108.3	C16—O6—C20	117.3 (7)
N1—C12—H12B	108.3		
			
C6—C1—C2—C3	0.3 (16)	C13—C14—C15—C16	−0.7 (17)
O1—C1—C2—C3	179.9 (9)	C14—C15—C16—C17	−0.7 (16)
C1—C2—C3—C4	−0.9 (17)	C14—C15—C16—O6	179.7 (10)
C2—C3—C4—C5	1.1 (17)	O6—C16—C17—C18	−178.1 (9)
C3—C4—C5—C6	−0.8 (17)	C15—C16—C17—C18	2.2 (15)
C2—C1—C6—C7	−179.6 (10)	C16—C17—C18—O5	179.9 (9)
O1—C1—C6—C7	0.8 (13)	C16—C17—C18—C13	−2.5 (15)
C2—C1—C6—C5	0.0 (14)	C14—C13—C18—O5	179.0 (8)
O1—C1—C6—C5	−179.6 (9)	C12—C13—C18—O5	−3.5 (12)
C4—C5—C6—C1	0.3 (15)	C14—C13—C18—C17	1.1 (13)
C4—C5—C6—C7	179.8 (10)	C12—C13—C18—C17	178.6 (9)
C1—C6—C7—C8	−0.6 (14)	O4—C11—N1—C12	−3.4 (13)
C5—C6—C7—C8	179.9 (10)	C10—C11—N1—C12	178.1 (8)
C1—C6—C7—O3	−177.3 (8)	C13—C12—N1—C11	83.3 (11)
C5—C6—C7—O3	3.2 (14)	O2—C9—O1—C1	177.8 (9)
O3—C7—C8—C9	177.7 (9)	C8—C9—O1—C1	2.3 (15)
C6—C7—C8—C9	1.1 (14)	C2—C1—O1—C9	178.7 (10)
O2—C9—C8—C7	−177.0 (11)	C6—C1—O1—C9	−1.7 (13)
O1—C9—C8—C7	−2.0 (15)	C8—C7—O3—C10	−7.3 (12)
O3—C10—C11—O4	164.9 (8)	C6—C7—O3—C10	169.5 (8)
O3—C10—C11—N1	−16.4 (11)	C11—C10—O3—C7	−160.9 (7)
N1—C12—C13—C14	2.3 (14)	C17—C18—O5—C19	−2.8 (13)
N1—C12—C13—C18	−175.0 (8)	C13—C18—O5—C19	179.4 (10)
C18—C13—C14—C15	0.5 (15)	C17—C16—O6—C20	1.9 (14)
C12—C13—C14—C15	−176.8 (10)	C15—C16—O6—C20	−178.4 (11)

### N-(2,4-Dimethoxybenzyl)-2-[(2-oxo-2H-chromen-4-yl)oxy]acetamide (II) Hydrogen-bond geometry (Å, º)

**Table:**

*D*—H···*A*	*D*—H	H···*A*	*D*···*A*	*D*—H···*A*
N1—H1···O3	0.86	2.31	2.669 (2)	105
C14—H14···N1	0.93	2.59	2.923 (2)	101
N1—H1···O4^i^	0.86	2.09	2.900 (2)	156
C3—H3···O5^ii^	0.93	2.49	3.419 (2)	175
C5—H5···O4^i^	0.93	2.43	3.307 (2)	157
C15—H15···O4^iii^	0.93	2.51	3.399 (2)	160

Symmetry codes: (i) −*x*+1, *y*+1/2, −*z*+1; (ii) −*x*+2, *y*+1/2, −*z*+1; (iii) −*x*, *y*+1/2, −*z*+1.
